# Clinical effectiveness and cost-effectiveness of ambulatory heart failure nurse-led services: an integrated review

**DOI:** 10.1186/s12872-022-02509-9

**Published:** 2022-02-22

**Authors:** Andrea Driscoll, Lan Gao, Jennifer J. Watts

**Affiliations:** 1 School of Nursing and Midwifery, Deakin University, 1 Gheringhap Street, Geelong, VIC 3220 Australia; 2 Department of Cardiology, Austin Health, Studley Rd, Heidelberg, VIC 3081 Australia; 3 School of Health Economics, Deakin University, 1 Gheringhap Street, Geelong, VIC 3220 Australia

**Keywords:** Economic evaluation, Cost-effectiveness, Heart failure, Nursing, Clinics, Remote monitoring, Cardiac failure, Cost benefit analysis

## Abstract

**Background:**

Globally the burden of heart failure is rising. Hospitalisation is one of the main contributors to the burden of heart failure and unfortunately, the majority of heart failure patients will experience multiple hospitalisations over their lifetime. Considering the high health care cost associated with heart failure, a review of economic evaluations of post-discharge heart failure services is warranted.

**Aim:**

An integrated review of the economic evaluations of post-discharge nurse-led heart failure services for patients hospitalised with acute heart failure.

**Methods:**

Electronic databases were searched using EBSCOHost: CINAHL complete, Medline complete, Embase, Scopus, EconLit, Global Health, and Health source (Consumer and Nursing/Academic) for published articles until 22nd June 2021. The searches focussed on papers that examined the cost-effectiveness of nurse-led clinics or telemonitoring involving nurses to follow-up patients after hospitalisation for acute heart failure. GRADE criteria and CHEERS checklist were used to determine the quality of the evidence and the quality of reporting of the economic evaluation.

**Results:**

Out of 453 studies identified, eight studies were included: four in heart failure clinics and four in telemonitoring programs. Five of the articles were cost-effectiveness analyses, one a cost comparison and two studies involved economic modelling The GRADE criteria were rated as high in five studies. In which, four studies examined the cost-effectiveness of telemonitoring programs. Based on the CHEERS checklist for reporting quality of economic evaluations, the majority of economic evaluations were rated between 86 and 96%. All the studies found the intervention to be cost-effective compared to usual care with Incremental Cost Effectiveness Ratios ranging from $18 259 (Canadian dollars)/life year gained to €40,321 per Quality Adjusted Life Years gained.

**Conclusion:**

Nurse-led heart failure clinics and telemonitoring programs were found to be cost-effective. Certainly, this review has shown that heart failure clinics and telemonitoring programs do represent value for money with their greatest impact and cost savings through reducing rehospitalisations.

**Supplementary Information:**

The online version contains supplementary material available at 10.1186/s12872-022-02509-9.

## Introduction

Hospital admissions for heart failure are predicted to rise substantially over the next decade placing pressure on the health care system leading to an increase in health care costs. Globally the burden of heart failure is escalating with over 23 million people worldwide diagnosed with heart failure [1]. The lifetime risk of developing heart failure is also high ranging from 30 to 42% in males and 32 to 39% in females [2]. The majority of these patients will experience not one but several hospitalisations for acute heart failure over their lifetime. The prognosis of heart failure is poor with a 10% in-hospital mortality rate from acute heart failure, post-discharge 30% mortality rate within one year [3, 4], and 20–25% will be rehospitalised within one month [5, 6].

The high rate of hospitalisations account for a large proportion of health care expenditure on heart failure. In Europe, 1–3% of total health care expenditure has been attributable to heart failure [7]. This includes inpatient, outpatient, pharmacotherapy, community services and medical devices. In the USA these costs are predicted to almost double over the next 15 years with 2017 costs at $30 billion (USD) [8] increasing to $53 billion (USD) by 2030 [9].

Given the significant financial and disease burden associated with heart failure, post discharge support is vital to reduce costs and improve outcomes in heart failure patients. Telemonitoring programs and HF clinics have been shown to reduce the risk of rehospitalisation by 36% [10] and 9% respectively [11]. However, the economic value of these programs has not been evaluated, emphasising the need for a review of the economic evidence. Economic evaluations provide a systematic approach to inform decision-makers about the most efficient use of their resources and provides an analysis of which program is more cost-effective. This systematic approach provides transparency, objectivity and accountability in decision-making [12]. The aim of this paper is to provide an integrated review of the economic evaluations of ambulatory nurse-led heart failure clinics and telemonitoring programs for patients diagnosed with heart failure.

## Methods

We conducted a review of economic evaluations of nurse-led heart failure clinics and telemonitoring follow-up for patients discharged from hospital with heart failure.

### Study eligibility

All articles that involved an economic evaluation of a nurse-led heart failure service in telemonitoring programs or clinic-based programs that included cost-effectiveness analysis, cost utility analysis, cost benefit analysis, cost-consequent analysis, cost-minimisation analysis and/or simulation modelling, were included in the review. All articles were restricted to English. There was no restriction on the year of publication or type of original study. Economic evaluations of pharmaceuticals and cardiac technologies were excluded, as the focus was on nurse-led heart failure services involving telemonitoring programs and heart failure clinics.

### Literature search

The Preferred Reporting System for Systematic Reviews and Meta-Analysis (PRISMA) [12] strategy was followed to ensure systematic selection of studies. A comprehensive search of the literature was undertaken on June 22nd 2021 using the database platform of EBSCOHost of the following databases: CINAHL complete, Medline complete, Embase, Scopus, EconLit, Global Health, and Health source (Consumer and Nursing/Academic). The searches focussed on papers that examined the cost-effectiveness of clinics or telemonitoring to manage patients following hospitalisation for acute heart failure. We also reviewed the reference lists of relevant papers, searched websites and conference papers. The following search terms using Boolean phrases were: “failure* OR incompet* OR insufficien*” AND “card* OR heart* OR myocard*”, AND: “Cost-effectiveness analysis” or “economic analysis” or “cost–benefit analysis” or “cost-utility analysis” or “cost-consequent analysis” or “cost-minimisation analysis” or “simulation modelling” or “markov modelling” or “economic evaluation”. Additional search terms included: “telemonitoring, telephone support or telehealth” OR “ambulatory care” or “outpatient clinic” or “clinic”.

### Quality assessment

The Consolidated Health Economic Evaluation Reporting Standards (CHEERS) checklist [13] was used to determine the reporting quality of economic evaluations. The checklist comprises of 24 items that are rated as ‘Yes’ meeting the criteria or ‘No’ did not meet the criteria or ‘NA’ not applicable. Any items that were partially met were also scored as ‘No’. The reporting quality of each study was then expressed as a percentage of the proportions of items that met the criteria for reporting economic evaluations. Studies that scored < 50% were rated as poor quality of reporting, 51–75% as moderate quality of reporting and > 75% were considered high quality of reporting economic evaluations in terms of meeting the reporting standards.

The Grading of Recommendations Assessment, Development and Evaluation (GRADE).

System [14] criteria were used to evaluate the quality of evidence for the estimate of effect, in each study. The quality of clinical evidence was taken from the original study that the economic evaluation was based on. Studies were rated as high quality when further research is unlikely to change the estimate of effect; moderate quality when further research may be likely to change the estimate of effect; low quality when further research is very likely to change the estimate of effect; and very low quality when there is a large degree of uncertainty about the estimate of the effect [14]. Based on the GRADE approach [14], randomised trails are rated as high quality evidence and observational studies as low quality evidence. There are five factors that can downgrade the quality of evidence for a randomised trial: study limitations, imprecision, inconsistency of results, publication bias and indirectness of evidence [14]. There are three factors that can improve the quality of evidence for observational studies: large degree of effect, confounders may minimise the effect and a dose response [14]. Additional details about the application of GRADE to evaluate quality of evidence is from a published article by Guyatt [14].

## Results

**Fig. 1:**
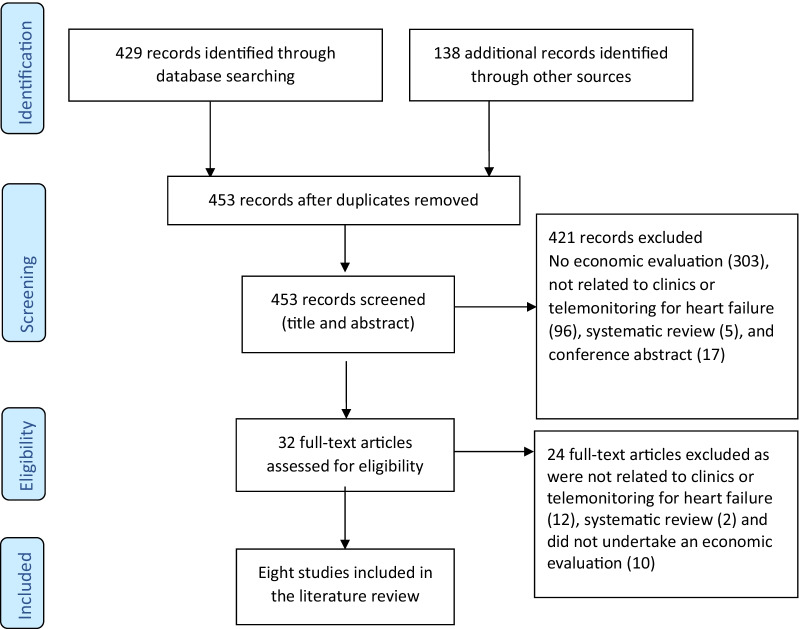
PRISMA flow diagram of literature review process

A total of 567 peer reviewed articles were identified of which 453 were selected for further review based on relevance of title and abstract screening (Fig. 1). A total of eight peer-reviewed articles were included in the review. Four articles investigated the cost-effectiveness of heart failure clinics and the other four articles telemonitoring. Five of the articles were cost-effectiveness analyses, one a cost comparison and two studies involved economic modelling; one study over 30 years [15] and another over a lifetime time horizon [16]. Three studies were an analysis conducted alongside an RCT [17–19], one study was a cohort design [20], one based on a pre and post-test design [21] and three used data from meta-analyses [15, 16, 22]. All of the studies are summarised in Table 1. The ICER varied across the studies ranging from $18,259 (Canadian dollars)/life year gained to €40,321 per QALY gained [15–20, 22]. All of the studies found a nurse-led heart failure clinic or telemonitoring program to be cost-effective compared to usual care.

**Table 1:** Summary of economic evaluations, CHEERS score and GRADE quality of evidence

References	Participants, country and time horizon	Type of study and comparators	Inputs	Effectiveness measure	Type of economic evaluation and outcome	CHEERS quality of reporting score (13)	GRADE quality of evidence* (14)
*HF clinic*							
Turner [19]	1163 patients with CHD or HF in 20 primary care practice clinics with follow-up over 12 months Country: United Kingdom	Cluster RCT Patients seen in GP practice (n = 658) versus patients seen weekly in a HF nurse clinic (n = 505)	Costs of intervention, medications, hospital appointments, travel costs and hospitalisation costs	QALYs measured by EuroQol	Cost-effectiveness HF nurse clinics were cost-effective at an ICER of £13 158 per QALY gained UK pounds	76%	High
Wijeysundera [20]	16,443 patients discharged from hospital with HF with follow-up over 12 years Country: Canada	Cohort study Patients seen in a usual care clinic versus hypothetical cohort managed in a multidisciplinary HF clinic Based on two clinic visits/year	Costs of clinics from existing clinic, staffing costs and overheads, costs of diagnostic tests, medications, hospitalisations, emergency department presentations Transition probabilities from meta-analyses	Life expectancy as measured in the EFFECT study [12]	Cost-effectiveness HF clinics were cost-effective with an ICER of $18,259/life-year gained Canadian dollars	86%	Moderate
Craswell [21]	HF patients seen in an outpatient clinic at one hospital with follow-up over 12 months Country: Australia	Pre and post design Patients seen in usual Cardiology clinic (n = 75) versus patients seen fortnightly in a NP titration clinic (n = 139)	Cost of clinic, personnel salary, and consumables from local service for NP clinic Usual Cardiology clinic costs from a national database. Includes same costs as for NP clinic but it is unclear if Cardiologist salary was included	No measure	Cost comparison Total cost per clinic visit was lower in NP titration clinic ($316) versus usual Cardiology clinic ($480) Total cost of NP titration clinic was $136 464 versus total cost of Cardiology clinic $153 456 Australian dollars	67%	Very low
Blum [16]	HF patients recently discharged from hospital. Modelling over a lifetime Country: USA	Meta-analysis Standard care versus disease management clinic versus home visits versus case management	Mortality and rehospitalisation modelled from a retrospective cohort of 3 million medicare pateints. Costs of hospitalisations, clinics, staffing, overheads, diagnostic tests, and medications were included. Cost of interventions were from RCTs. Transition probabilities from meta-analyses	QALYs as measured in the EPHESUS trial [23]	Cost-effectiveness with decision analytic decision model Home visits were cost-effective with an ICER of $19,570 per QALY gained All 3 interventions were cost-effective at a WTP of $50,000 US dollars	96%	High
*HF telemonitoring programs*							
Hebert [17]	406 HF patients from outpatient clinics in Harlem, NY were followed up for 12 months Country: USA	RCT 203 HF patients assigned to usual care versus 203 HF patients to nurse telephone follow-up including one clinic visit	Costs of intervention, transportation costs, cost of salaries and overheads, cost of hospitalisations, clinics and ED presentations, patient diaries determined time in medical appointments, informal carer costs	QALYs measured by EuroQol-5D	Cost-effectiveness analysis Nurse managed group was cost-effective with an ICER of $17,543 per QALY gained US dollars	91%	High
Klersy [22]	HF patients from the community and followed up for 12 months Country: multiple countries	Based on meta-analysis of 21 RCTs of remote monitoring versus usual care	Costs of hospitalisations	QALYs calculated as survival gain multiplied by utility gain Utilities were taken from published trials	Cost-effectiveness Cost differences between the two groups ranged from €300- €1000 favouring RPM with a QALY gain of 0.06 Euros	91%	High
Boyne [18]	HF patients from outpatient clinics in 3 hospitals and were followed up for 12 months Country: Netherlands	RCT Total of 382 HF patients were randomised to telemonitoring (n = 197) versus usual care (n = 185)	Cost diary provided data on home care costs, outpatient visits to various healthcare providers and GP visits. Also hospitalisations, ED presentations, and medication costs	QALYs measured by EQ-5D	Cost-effectiveness Telemonitoring was cost-effective with an ICER of €40,321 per QALY gained. However the probability of telemonitoring being cost-effective at a threshold of €50,000 was 48% Euros	86%	High
Thokala [15]	HF patients recently discharged from a HF hospitalisation were followed up for six months Time horizon was over 30 years Country: United Kingdom	Network meta-analysis of 21 RCTs (6317 HF patients) comparing usual care with telemonitoring structured telephone support (STS) human-to-human or STS human-to-machine	Costs of the intervention, hospitalisation, and usual care	QALYs were taken from four RCTs of the different interventions	Cost-effectiveness with Markov model Telemonitoring was cost-effective, compared to usual care, at an ICER of £11,873 per QALY gained UK pounds	86%	High

^*^GRADE of evidence [13]: high quality when further research was unlikely to change the estimate of effect; moderate quality when further research may be likely change the estimate of effect; low quality when further research is very likely to change the estimate of effect; and very low quality when there is a large degree of uncertainty about the estimate of effect

### Heart failure clinic economic evaluations

Four studies undertook an economic evaluation of a heart failure clinic. One study evaluated a nurse-led titration clinic [21], another investigated a nurse-led clinic in primary care [19], another study compared three interventions (clinic vs home visits vs case management) [16] and the other study performed a cost-effectiveness analysis of specialised multidisciplinary heart failure clinics [20]. None of the studies were from the same country.

One study that rated high quality of reporting on the CHEERS checklist with 86% (Additional file 1: Table S1a), undertook a modelled cost-effectiveness analysis of a hypothetical clinic based on data from various sources [20]. Transition probabilities and outcomes were taken from a multicentre RCT for the usual care arm and meta-analysis for the multidisciplinary heart failure clinic intervention arm. Another study conducted an economic evaluation alongside a cluster RCT of a nurse-led clinic in primary care compared to a GP clinic for patients diagnosed with coronary heart disease and heart failure [19]. According to the CHEERS checklist it rated as 76% (Additional file 1: Table S1b). This nurse-led clinic showed an increase in QALY of 0.03/year and an increase in health care costs of £425. The clinics generated an ICER of £13,158/QALY compared to usual care [19]. Wjeysundera et al. [20] did not use QALYs as an outcome measure but looked at life-years gained. They found the predicted life expectancy of heart failure clinic patients to be 3.91 years compared to 3.21 years for standard care [20]. The 12-year cumulative cost per patient in the heart failure clinic group was $66,532 (Canadian dollars (CAD)) vs $53,638 (CAD) in the usual care group. The ICER was $18,259 (CAD)/life-year gained [20]. It is difficult to generalise the results of both these studies due to two main limitations. The study by Wjeysundera and colleagues [20] used a time horizon of 12 years with each patient having two appointments within the clinic annually. This assumption is likely to be an under-estimation as patients are unlikely to visit the same hospital-based clinic six monthly for 12 years. The population in the study by Turner and colleagues [19] included people with coronary heart disease (CHD) or heart failure, making it difficult to attribute costs separably to the heart failure population.

A study by Blum and colleagues [16] was rated as a high quality report of an economic evaluation with a CHEERS score of 96% (Additional file 1: Table S1c). The economic evaluation used a decision analytic microsimulation model with a lifetime time horizon. They compared three different interventions involving heart failure nurses (clinic, home visits and case management) to usual care over a lifetime. Their input parameters were sourced from systematic reviews of the different interventions. They found that home visits compared to usual care were the most cost-effective with an ICER of $19,570 (US dollars) per QALY gained. However, all of the three interventions, including clinics, were cost-effective compared to usual care at a WTP threshold of $50,000 per QALY gained. However, only the ICER associated with home visits compared to usual care was reported. Unfortunately, no head-to-head comparisons were reported such as clinic versus home visits or case management versus home visits.

Craswell et al. [21] undertook an economic analysis of a heart failure nurse practitioner medication titration clinic for patients diagnosed with heart failure. The study design was a cost-comparison of the nurse-led clinic compared to a medical-led clinic in one hospital. This study was rated as 67% on the CHEERS checklist (Additional file 1: Table S1d). The case level costs associated with visits to the usual care medical clinic were stated as ‘not available’, instead the authors modelled medical clinic costs based on average costs reported in the National Hospital Cost Data Collection [23]. As a single centre study, it would have been more accurate to obtain hospital specific clinical costing from their hospital clinical costing unit. Authors state that all costs were adjusted for inflation but the rate of inflation was not stated. The clinic in this study was aimed specifically at the titration of medications in heart failure patients. However, medication costs were not included in the economic evaluation. As no outcome measures were included in the analysis, the study method was a cost comparison not a cost-effective analysis as reported by the authors. Craswell and colleagues [21] found that the total cost per patient attending the nurse practitioner clinic was $41 less than the cost per patient attending usual care medical staff clinic [21]. However, it is uncertain where these cost savings came from as the nurse practitioner clinic had a higher number of clinic visits but lower cost per visit.

The GRADE criteria were applied to each of the studies [14]. The GRADE criteria for the study by Turner and colleagues [19] was rated as high as their economic evaluation was based on a large multicentre cluster randomised controlled trial (RCT). Blum and colleagues [16] was rated as high as their economic data were sourced from meta-analyses and RCTs. The study by Wijeysundera and colleagues [20] was rated as moderate as their quality of evidence was based on a cohort study but the sample involved 16,443 patients. So the quality of evidence was upgraded from low. The heart failure clinics were hypothetical and the data were based on systematic reviews and meta-analyses. They estimated that on average patients had two clinic visits annually. This is an under-estimation of the number of visits as patients diagnosed with heart failure experience frequent exacerbations often requiring more than two clinic visits/year. Life expectancy was taken from a previous published study that followed up patients for 12 years [24]. The study by Craswell and colleagues [21] was rated as very low due to the pre and post study design, data analysis and issues of uncertainty so their GRADE criteria was downgraded from low.

### Economic evaluations of telemonitoring

There are two main types of telemonitoring in heart failure. One is a structured telephone support system where the patient dials into an automated voice activation system. Patients then enter their responses to questions via a touch type phone eg 1 = yes; 2 = no and they can also enter their weight. The other type of system is fully automated so the patient dials into the remote system. In their home they have a blood pressure machine and a set of weighing scales that are connected to the system. When instructed the patient will put on the blood pressure cuff and their blood pressure will be measured automatically and they will also stand on the scales when instructed and their weight will be transferred automatically into the monitoring system. Some systems also have several education modules that the patient can listen to for example fluid restriction or heart failure disease or self-management [25].

The literature search identified four economic evaluations involving telemonitoring. Two were undertaken using data from meta-analyses [15, 22] and two were conducted alongside an RCT [17, 18]. Based on the CHEERS checklist, the reporting quality of these studies ranged from 86 to 91% (Additional file 1: Table S2a–d). One study [18] was assigned a lower CHEERS score due to lack of discussion of the effects of uncertainty and distribution of parameters (Additional file 1: Table S2a). Thokala and colleagues [15] was also assigned a CHEERS reporting score of 86% due to lack of information about underlying assumptions of their model and methods used to estimate costs and conversion (Additional file 1: Table S2b). When the GRADE criteria were applied, all of the studies were rated as a high quality of evidence. These studies were conducted alongside an RCT [17, 18] or based on a network meta-analysis [15, 22]. None of the studies were from the same country. The economic evaluation by Klersy and colleagues [21] was based on a meta-analysis where the country of origin of the randomised controlled trial varied.

One cost-effectiveness analysis was based on a meta-analysis of 21 randomised controlled trials on telemonitoring compared with usual care [22] and was rated with a high quality of reporting on the CHEERS checklist with 91% (Additional file 1: Table S2c). Usual care involved one visit to an outpatient clinic, general practitioner office or a visit to the Emergency Department. A telephone monitoring approach included regular structured telephone contact and referral of symptoms. The cost-effectiveness analysis was built on efficacy data from 17 RCTs. There was no justification as to why four studies were excluded from the cost-effectiveness analysis. Also, the analysis combined several interventions under the term of ‘remote patient monitoring’ and then compared it to usual care rather than deconstructing the term into separate interventions and comparing each type of intervention to usual care. Cost utility analysis using QALYs was undertaken from a payer perspective with 12 months of follow up. Utilities were retrieved from a RCT by Hebert and colleagues [17] and scores were 0.612 for usual care and 0.662 for remote patient monitoring (RPM) groups. However, the study by Herbert and colleagues [17] was in African-American and Hispanic heart failure patients attending a clinic in Harlem. Klersy et al. [22] have assumed that the utilities from a disadvantaged population [17] would be applicable to their patient population with middle to high incomes. In the decision analytic model by Klersy and colleagues [22] the transition probabilities were taken from their meta-analysis. There were two transition states: no hospitalisation or hospitalised for heart failure over a 12- month time horizon. There was no reporting of an additional arm in their model to account for mortality and the transition probability of background mortality. The costs of hospitalisation were based on diagnostic related groups. The cost differences between the two groups ranged from €300- €1000 favouring RPM. There was a QALY gain of 0.06 suggesting that remote patient monitoring was a dominant strategy over usual care.

Unlike Klersy and colleagues [22], Thokala et al. [15] compared different types of telemonitoring and usual care over 12 months. The interventions were: structured telephone support (human-to-human and human-to-machine) and telemonitoring, and usual care. Each of the interventions were compared with usual care and also against each other. A Markov model was developed with two transition states: alive and dead. Transition probabilities for risk of readmission was sourced from a meta-analysis by Klersy and colleagues [22]. Mortality probabilities were taken from CHARM study [26]. Base case cost-effectiveness suggests that telemonitoring during office hours compared to usual care was the most cost effective at a threshold of £20,000/QALY. Sensitivity analyses using higher costs of usual care and each intervention did not change the ICER. Health Related Quality of Life was based on QALYs for usual care which was sourced from previous studies with an average QALY of 0.57–0.6. Costs included: cost of service after initial discharge only, costs of usual care, and readmission costs. Hospitalisation costs were based on DRGs. Telemonitoring was the dominant strategy with an ICER of £11,873/QALY compared to usual care. Structured telephone support via human to human interface had an ICER of £228 035/QALY compared to telemonitoring. Usual care was the dominant strategy over structured telephone support via human to machine interface.

Hebert and colleagues [17] undertook a cost-effectiveness study alongside a RCT of a telephone support system for patients with heart failure attending an outpatient clinic from four hospitals in Harlem. They also received a high quality of reporting based on the CHEERS checklist with 91% (Additional file 1: Table S2d). QALYs were measured by EQ-5D at baseline and 12 months. A payer perspective was used with a 12-month time horizon. The nurse-led telephone support system was cost-effective with an ICER of $17,543 per QALY compared to usual care. Sensitivity analysis showed no difference. However, 85% of participants enrolled in the trial were black/Hispanic from a low SES area of New York so the results may not be generalisable to the general population.

The final economic evaluation involving telemonitoring was based on an RCT of 382 heart failure patients randomised to telemonitoring or usual care and followed up for 12 months from three hospitals [18]. The usual care group received four outpatient clinic visits over 12 months and the telemonitoring group received two outpatient clinic visits and telemonitoring with daily surveillance and monitoring of symptoms over 12 months. All patients were asked to record in a cost diary, the number of GP visits, specialist follow-up and multidisiciplinary team appointments such as physiotherapy, heart failure nurse, pharmacists and so on. Inpatient costs were taken from the individual hospital costing system. ED presentations and rehospitalisation costs were from a national costing manual. Cost of medications were based on the Dutch Pharmacotherapeutic Compass. EQ-5D was used to elicit utilities. Boyne and colleagues found that at a WTP of €50,000 the probability of telemonitoring being cost-effective was 48% [18]. Overall, the ICER of telemonitoring versus usual care was €40,321 per QALY gained.

The uncertainty analysis showed that in one hospital, telemonitoring was not cost-effective but in the other two sites, it was. The ICERs at two sites were €22,216 and €23,051 per QALY gained compared to the ICER at the other site of €55,256 per QALY gained. There was no significant difference in QALYs or total costs between telemonitoring and usual care.

## Discussion

This review summarises economic evaluations of nurse-led heart failure clinics and telemonitoring programs for patients diagnosed with heart failure. The quality of reporting of economic evaluations was assessed using the CHEERS checklist and quality of evidence was assessed according to the GRADE criteria. Economic evaluation methods differed across the included studies ranging from full economic evaluation with ICER reported to cost comparisons. These differences were reflected in the quality assessment tools that we used to compare studies [13, 14].

Economic evaluations of nurse-led telemonitoring programs were based on a high quality of evidence mainly due to evaluations being conducted alongside large, multicentre RCTs or based on large meta-analyses. Studies involving nurse-led heart failure clinics mainly sourced their input parameter data from observational or single centre studies. Economic evaluations of heart failure clinics need to be conducted alongside a multicentre RCT. However, heart failure clinics are now entrenched within usual care so it is unlikely that large multicentre RCTs will be conducted.

Nurse-led heart failure clinics in general were found to be cost-effective but heterogeneity in clinic type/service provided would likely impact on the cost-effectiveness. The findings from this review are consistent with the outcomes literature that has found them to be clinically effective [11, 27, 28]. Cost savings from heart failure clinics are likely to be from reduced rehospitalisations at 7 and 30 days. One study modelled three interventions over a lifetime: clinic, home visits, case management and usual care [16]. They found all three interventions to be cost-effective compared to usual care which is also reflective of the evidence of these interventions on reducing rehospitalisations [27–29].

Telemonitoring programs were cost-effective in three of the studies but only a 48% probability of being cost-effective in a fourth study [18]. Several large RCTs have not found telemonitoring to be clinically effective compared to usual care [30, 31] indicating that more evidence is warranted. Two of the economic evaluations used data from a meta-analysis so depending on which meta-analysis was used, it would have an impact on the cost-effectiveness of telemonitoring [32]. The main cost driver in the economic evaluations was a reduction in rehospitalisations. In all of the telemonitoring evaluations, the usual care groups also received follow up in an outpatient clinic [15, 17, 18, 22]. There may have been a greater difference in ICER if the usual care groups did not include a clinic review. One study combined structured telephone support studies with telemonitoring studies and overall found them to be cost-effective [22]. Structured telephone support only was also found to be cost-effective (ICER $17,543/QALY) [17]. However, in a head-to-head comparison of structured telephone support to telemonitoring, telemonitoring was the dominant economic strategy [15].

Transferability of the ambulatory HF nurse-led services in this review is difficult due to heterogeneity of country of the study, different currencies used and year of undertaking the studies varied. Although this review focussed on nurse-led clinics and telemonitoring, there was significant heterogeneity between studies which would impact on transferability between health settings. The measure of effectiveness also varied with some studies using QALYs [15, 16, 18, 19], one study used life expectancy [12] and another survival gain [22].

## Limitations

There were two limitations associated with this review. Firstly, a quantitative synthesis of the literature was not undertaken due to the low number of included studies and heterogeneity of study design. Secondly, the review of economic evaluations of heart failure clinics and telemonitoring programs was taken by one reviewer. Any uncertainty in study design, method, results interpretation or grading of studies were discussed with the other two authors.

## Conclusion

Heart failure has a high burden of disease with the main driver of health care costs being rehospitalisation. Economic evaluations of nurse-led heart failure services have shown the services to be cost-effective compared to a usual care comparator. From a health system perspective this information can inform policy direction about provision of services that represent ‘value for money’. Certainly, this review has shown that nurse-led heart failure clinics and telemonitoring programs do represent value for money with their greatest impact on cost savings in reducing rehospitalisation.

## Supplementary Information

**Additional file 1**. A critical appraisal of the reporting of economic evaluations of Heart Failure programs based on the CHEERS checklist

## Data Availability

All data generated or analysed during this study are included in this published article and its Additional file 1.

## Abbreviations

CAD: Canadian dollars
CHD: Coronary heart disease
CHEERS: Consolidated health economic evaluation reporting standards
ED: Emergency department
GRADE: Grading of recommendations assessment, development and evaluation
ICER: Incremental cost effectiveness ratio
PRISMA: Preferred reporting system for systematic reviews and meta-analysis
QALY: Quality adjusted life years
RCT: Randomised controlled trial
