# Supplementary material for: Clinical effectiveness and cost-effectiveness of ambulatory heart failure nurse-led services: an integrated review
Source: BMC Cardiovasc Disord. 2022 Feb 22;22:64. doi: 10.1186/s12872-022-02509-9 (PMC8862539; doi:10.1186/s12872-022-02509-9)
Supplement: Supplementary file 1 — Additional file 1. A critical appraisal of the reporting of economic evaluations of Heart Failure programs based on the CHEERS checklist [file 12872_2022_2509_MOESM1_ESM.docx]

# SUPPLEMENTARY APPENDIX

# CHEERS CHECKLIST

**CHEERS checklist (13) —Critical appraisal of the reporting of economic evaluations of Heart Failure programs.**

**Supplementary Table 1: Economic evaluations of HF clinics**

**Supplementary Table 1a: Critical appraisal of Turner et al. 2008 (19)**

| **Section/item** | **Item no.** | **Recommendation** | **Reported** | **Discussion** |
| --- | --- | --- | --- | --- |
| **Title and abstract** | | | | |
| Title | 1 | Identify the study as an economic evaluation, or use more specific terms such as “cost-effectiveness analysis” and describe the interventions compared. | YES | No comparator in the title. No economic perspective. |
| Abstract | 2 | Provide a structured summary of objectives, perspective, setting, methods (including study design and inputs), results (including base-case and uncertainty analyses), and conclusions. | YES | No uncertainty analysis |
| **Introduction** | | | | |
| Background and objectives | 3 | Provide an explicit statement of the broader context for the study. | YES | Describes CHD and HF. Secondary prevention in primary care. |
|  |  | Present the study question and its relevance for health policy or practice decisions. | YES |  |
| **Methods** | | | | |
| Target population and subgroups | 4 | Describe characteristics of the base-case population and subgroups analysed including why they were chosen. | YES | Table 1  Two groups are unequal so 75% of sample have CHD and 25% HF  Also more patients in UC than intervention  Two arms statistical difference at baseline. Brief data on co-morbidities |
| Setting and location | 5 | State relevant aspects of the system(s) in which the decision(s) need(s) to be made. | YES | Primary care |
| Study perspective | 6 | Describe the perspective of the study and relate this to the costs being evaluated. | NO | Not stated |
| Comparators | 7 | Describe the interventions or strategies being compared and state why they were chosen. | YES | 658 pts in UC randomised to  505pts randomised to intervention of a nurse-led clinic in GP practice |
| Time horizon | 8 | State the time horizon(s) over which costs and consequences are being evaluated and say why appropriate. | YES | 12 months. No justification for 1 year |
| Discount rate | 9 | Report the choice of discount rate(s) used for costs and outcomes and say why appropriate. | YES | No discount |
| Choice of health outcomes | 10 | Describe what outcomes were used as the measure(s) of benefit in the evaluation and their relevance for the type of analysis performed. | YES | QALY, readmissions and mortality |
| Measurement of effectiveness | 11a | *Single study–based estimates*: Describe fully the design features of the single effectiveness study and why the single study was a sufficient source of clinical effectiveness data. | YES | Economic evaluation done alongside a cluster RCT involving 20 GP practices |
|  | 11b | *Synthesis-based estimates*: Describe fully the methods used for the identification of included studies and synthesis of clinical effectiveness data. | NA |  |
| Measurement and valuation of preference-based outcomes | 12 | If applicable, describe the population and methods used to elicit preferences for outcomes. | NA |  |
| Estimating resources and costs | 13a | *Single study–based economic evaluation*: Describe approaches used to estimate resource use associated with the alternative interventions. Describe primary or secondary research methods for valuing each resource item in terms of its unit cost. Describe any adjustments made to approximate to opportunity costs. | YES | Patient diary for travel costs  Medication costs from national database  Staff salaries from national database  Outpatient and GP clinic visits  Hospitalisation costs from NHS  QALY- EQ 5D at baseline and 12m |
|  | 13b | *Model-based economic evaluation***:** Describe approaches and data sources used to estimate resource use associated with model health states. Describe primary or secondary research methods for valuing each resource item in terms of its unit cost. Describe any adjustments made to approximate to opportunity costs. | NA |  |
| Currency, price date, and conversion | 14 | Report the dates of the estimated resource quantities and unit costs. Describe methods for adjusting estimated unit costs to the year of reported costs if necessary. Describe methods for converting costs into a common currency base and the exchange rate. | NO | No conversion, cost taken from year of enrolment |
| Choice of model | 15 | Describe and give reasons for the specific type of decision-analytic model used. Providing a figure to show model structure is strongly recommended. | NA |  |
| Assumptions | 16 | Describe all structural or other assumptions underpinning the decision-analytic model. | NA |  |
| Analytic methods | 17 | Describe all analytic methods supporting the evaluation. This could include methods for dealing with skewed, missing, or censored data; extrapolation methods; methods for pooling data; approaches to validate or make adjustments (e.g., half-cycle corrections) to a model; and methods for handling population heterogeneity and uncertainty. | YES | Positively skewed cost data was transformed to estimate mean costs for both interventions  Distributions and goodness of fit discussed  Cost-effectiveness acceptability curve for uncertainty. No mention of type of sensitivity analysis or which variables were manipulated |
| **Results** | | | | |
| Study parameters | 18 | Report the values, ranges, references, and if used, probability distributions for all parameters. Report reasons or sources for distributions used to represent uncertainty where appropriate. Providing a table to show the input values is strongly recommended. | NO | No discussion of uncertainty or sensitivity analysis |
| Incremental costs and outcomes | 19 | For each intervention, report mean values for the main categories of estimated costs and outcomes of interest, as well as mean differences between the comparator groups. If applicable, report incremental cost-effectiveness ratios. | YES | Table 5 main cost table. No mean difference done only mean cost and CI  QALYs in Table 6 |
| Characterizing uncertainty | 20a | *Single study–based economic evaluation*: Describe the effects of sampling uncertainty for estimated incremental cost, incremental effectiveness, and incremental cost-effectiveness, together with the impact of methodological assumptions (such as discount rate, study perspective). | NO | Uncertainty not addressed |
|  | 20b | *Model-based economic evaluation*: Describe the effects on the results of uncertainty for all input parameters, and uncertainty related to the structure of the model and assumptions. | NA |  |
| Characterizing heterogeneity | 21 | If applicable, report differences in costs, outcomes, or cost-effectiveness that can be explained by variations between subgroups of patients with different baseline characteristics or other observed variability in effects that are not reducible by more information. | NA |  |
| **Discussion** | | | | |
| Study findings, limitations, generalizability, and current knowledge | 22 | Summarize key study findings and describe how they support the conclusions reached. Discuss limitations and the generalizability of the findings and how the findings fit with current knowledge. | YES | Increase in QALY 0.03/yr and increase in cost of £425  ICER £13 158/QALY compared to control group |
| **Other** | | | | |
| Source of funding | 23 | Describe how the study was funded and the role of the funder in the identification, design, conduct, and reporting of the analysis. Describe other nonmonetary sources of support. | NO |  |
| Conflicts of interest | 24 | Describe any potential for conflict of interest among study contributors in accordance with journal policy. In the absence of a journal policy, we recommend authors comply with International Committee of Medical Journal Editors’ recommendations. | YES |  |
| **Total score** | | | | 76% (16/21) |

**Supplementary Table 1b: Critical appraisal of Wijeysundera et al. 2010 (20)**

| **Section/item** | **Item no.** | **Recommendation** | **Reported** | **Discussion** |
| --- | --- | --- | --- | --- |
| **Title and abstract** | | | | |
| Title | 1 | Identify the study as an economic evaluation, or use more specific terms such as “cost-effectiveness analysis” and describe the interventions compared. | YES | No comparator in the title. No economic perspective. |
| Abstract | 2 | Provide a structured summary of objectives, perspective, setting, methods (including study design and inputs), results (including base-case and uncertainty analyses), and conclusions. | YES | No uncertainty analysis |
| **Introduction** | | | | |
| Background and objectives | 3 | Provide an explicit statement of the broader context for the study. | YES |  |
|  |  | Present the study question and its relevance for health policy or practice decisions. | YES |  |
| **Methods** | | | | |
| Target population and subgroups | 4 | Describe characteristics of the base-case population and subgroups analysed including why they were chosen. | YES | Patients discharged from hospital with HF in 2005-16443 patients. See Table 4 |
| Setting and location | 5 | State relevant aspects of the system(s) in which the decision(s) need(s) to be made. | YES | Ambulatory care |
| Study perspective | 6 | Describe the perspective of the study and relate this to the costs being evaluated. | YES | Ministry of Health |
| Comparators | 7 | Describe the interventions or strategies being compared and state why they were chosen. | YES | The intervention was a hypothetical clinic.  Usual care: patients followed up in any type of clinic and not MD  Intervention: patients followed up in a MD HF clinic |
| Time horizon | 8 | State the time horizon(s) over which costs and consequences are being evaluated and say why appropriate. | YES | 12 years. Most patients don’t continue to attend a clinic for 12 years |
| Discount rate | 9 | Report the choice of discount rate(s) used for costs and outcomes and say why appropriate. | YES | 5% discount/year and adjusted for inflation to 2008 Canadian dollars |
| Choice of health outcomes | 10 | Describe what outcomes were used as the measure(s) of benefit in the evaluation and their relevance for the type of analysis performed. | YES | Mortality, hospitalisations, costs  No QALY |
| Measurement of effectiveness | 11a | *Single study–based estimates*: Describe fully the design features of the single effectiveness study and why the single study was a sufficient source of clinical effectiveness data. | YES | Used a hypothetical cohort for intervention. Took costs from a current clinic and inputted it into the intervention clinic |
|  | 11b | *Synthesis-based estimates*: Describe fully the methods used for the identification of included studies and synthesis of clinical effectiveness data. | NA |  |
| Measurement and valuation of preference-based outcomes | 12 | If applicable, describe the population and methods used to elicit preferences for outcomes. | NA |  |
| Estimating resources and costs | 13a | *Single study–based economic evaluation*: Describe approaches used to estimate resource use associated with the alternative interventions. Describe primary or secondary research methods for valuing each resource item in terms of its unit cost. Describe any adjustments made to approximate to opportunity costs. | YES | Costs for the intervention clinic were taken from a current clinic and estimated on average two clinics/year. Clinic costs in Table 2. Also included diagnostic test results  Costs for usual care taken from an administrative database.  Cost of hospitalisation used Resource Intensity Weighting (RIW).  Medication costs from national medication database and included only HF medications. |
|  | 13b | *Model-based economic evaluation***:** Describe approaches and data sources used to estimate resource use associated with model health states. Describe primary or secondary research methods for valuing each resource item in terms of its unit cost. Describe any adjustments made to approximate to opportunity costs. | NA |  |
| Currency, price date, and conversion | 14 | Report the dates of the estimated resource quantities and unit costs. Describe methods for adjusting estimated unit costs to the year of reported costs if necessary. Describe methods for converting costs into a common currency base and the exchange rate. | NO | No conversion, cost taken from year of enrolment |
| Choice of model | 15 | Describe and give reasons for the specific type of decision-analytic model used. Providing a figure to show model structure is strongly recommended. | NA |  |
| Assumptions | 16 | Describe all structural or other assumptions underpinning the decision-analytic model. | NO | Assumed costs of a current clinic related to a hypothetical model. Assumed an average of 2 clinic visits/yr. Most HF clinics see patients at least quarterly. |
| Analytic methods | 17 | Describe all analytic methods supporting the evaluation. This could include methods for dealing with skewed, missing, or censored data; extrapolation methods; methods for pooling data; approaches to validate or make adjustments (e.g., half-cycle corrections) to a model; and methods for handling population heterogeneity and uncertainty. | YES | One way deterministic sensitivity analysis was done. Also PSA with Monte Carlo simulation involving 10 000 trials. |
| **Results** | | | | |
| Study parameters | 18 | Report the values, ranges, references, and if used, probability distributions for all parameters. Report reasons or sources for distributions used to represent uncertainty where appropriate. Providing a table to show the input values is strongly recommended. | YES | RR and distributions described in Table 1.  Mortality rates taken from the EFFECT study to determine life-expectancy in UC group. In the intervention group, mortality taken form meta-analysis (ref 13). Survival curves for both groups were constructed. |
| Incremental costs and outcomes | 19 | For each intervention, report mean values for the main categories of estimated costs and outcomes of interest, as well as mean differences between the comparator groups. If applicable, report incremental cost-effectiveness ratios. | YES | See Table 5 |
| Characterizing uncertainty | 20a | *Single study–based economic evaluation*: Describe the effects of sampling uncertainty for estimated incremental cost, incremental effectiveness, and incremental cost-effectiveness, together with the impact of methodological assumptions (such as discount rate, study perspective). | YES | Sensitivity analysis showed results were robust. Varied medication costs and diagnostic tests and mortality rates |
|  | 20b | *Model-based economic evaluation*: Describe the effects on the results of uncertainty for all input parameters, and uncertainty related to the structure of the model and assumptions. | NA |  |
| Characterizing heterogeneity | 21 | If applicable, report differences in costs, outcomes, or cost-effectiveness that can be explained by variations between subgroups of patients with different baseline characteristics or other observed variability in effects that are not reducible by more information. | NA |  |
| **Discussion** | | | | |
| Study findings, limitations, generalizability, and current knowledge | 22 | Summarize key study findings and describe how they support the conclusions reached. Discuss limitations and the generalizability of the findings and how the findings fit with current knowledge. | YES | 12 year cumulative cost/patient in HF clinic was $66 532 vs $53 638 in UC group. ICER was $18 259/life-year gained |
| **Other** | | | | |
| Source of funding | 23 | Describe how the study was funded and the role of the funder in the identification, design, conduct, and reporting of the analysis. Describe other nonmonetary sources of support. | NO |  |
| Conflicts of interest | 24 | Describe any potential for conflict of interest among study contributors in accordance with journal policy. In the absence of a journal policy, we recommend authors comply with International Committee of Medical Journal Editors’ recommendations. | NO |  |
| **Total score** | | | | 86% (18/21) |

**Supplementary Table 1c: Critical appraisal of Craswell et al. 2017 (21)**

| **Section/item** | **Item no.** | **Recommendation** | **Reported** | **Discussion** |
| --- | --- | --- | --- | --- |
| **Title and abstract** | | | | |
| Title | 1 | Identify the study as an economic evaluation, or use more specific terms such as “cost-effectiveness analysis” and describe the interventions compared. | YES | No comparator in the title. No economic perspective.  Title say CE but methods are not CE more cost comparison |
| Abstract | 2 | Provide a structured summary of objectives, perspective, setting, methods (including study design and inputs), results (including base-case and uncertainty analyses), and conclusions. | NO | No uncertainty analysis. No results. No methods |
| **Introduction** | | | | |
| Background and objectives | 3 | Provide an explicit statement of the broader context for the study. | YES |  |
|  |  | Present the study question and its relevance for health policy or practice decisions. | YES |  |
| **Methods** | | | | |
| Target population and subgroups | 4 | Describe characteristics of the base-case population and subgroups analysed including why they were chosen. | YES | HF patients attending a NP clinic vs usual outpatient clinic |
| Setting and location | 5 | State relevant aspects of the system(s) in which the decision(s) need(s) to be made. | YES | Ambulatory care |
| Study perspective | 6 | Describe the perspective of the study and relate this to the costs being evaluated. | NO | Not stated |
| Comparators | 7 | Describe the interventions or strategies being compared and state why they were chosen. | YES | NP titration clinic vs usual outpatient clinic |
| Time horizon | 8 | State the time horizon(s) over which costs and consequences are being evaluated and say why appropriate. | YES | 1 year |
| Discount rate | 9 | Report the choice of discount rate(s) used for costs and outcomes and say why appropriate. | YES | Discount stated but not % or reason as only 1 year |
| Choice of health outcomes | 10 | Describe what outcomes were used as the measure(s) of benefit in the evaluation and their relevance for the type of analysis performed. | NO | No outcomes discussed and no QALYs |
| Measurement of effectiveness | 11a | *Single study–based estimates*: Describe fully the design features of the single effectiveness study and why the single study was a sufficient source of clinical effectiveness data. | YES | Pre and post test design: Usual care patients were selected from an outpatient cardiac clinic, seen by Cardiologist, pre NP clinic; Intervention patients attended the NP titration clinic in 2014 in one of 4 clinics |
|  | 11b | *Synthesis-based estimates*: Describe fully the methods used for the identification of included studies and synthesis of clinical effectiveness data. | NA |  |
| Measurement and valuation of preference-based outcomes | 12 | If applicable, describe the population and methods used to elicit preferences for outcomes. | NA |  |
| Estimating resources and costs | 13a | *Single study–based economic evaluation*: Describe approaches used to estimate resource use associated with the alternative interventions. Describe primary or secondary research methods for valuing each resource item in terms of its unit cost. Describe any adjustments made to approximate to opportunity costs. | YES | Cost of clinics over 2014, cost of outpatient care from NHCCC |
|  | 13b | *Model-based economic evaluation***:** Describe approaches and data sources used to estimate resource use associated with model health states. Describe primary or secondary research methods for valuing each resource item in terms of its unit cost. Describe any adjustments made to approximate to opportunity costs. | NA |  |
| Currency, price date, and conversion | 14 | Report the dates of the estimated resource quantities and unit costs. Describe methods for adjusting estimated unit costs to the year of reported costs if necessary. Describe methods for converting costs into a common currency base and the exchange rate. | NO | UC 2012 costs. No discussion of costs converted to 2014. Method not stated. Intervention cost taken from 2014 |
| Choice of model | 15 | Describe and give reasons for the specific type of decision-analytic model used. Providing a figure to show model structure is strongly recommended. | NA |  |
| Assumptions | 16 | Describe all structural or other assumptions underpinning the decision-analytic model. | NA |  |
| Analytic methods | 17 | Describe all analytic methods supporting the evaluation. This could include methods for dealing with skewed, missing, or censored data; extrapolation methods; methods for pooling data; approaches to validate or make adjustments (e.g., half-cycle corrections) to a model; and methods for handling population heterogeneity and uncertainty. | NO | No data analysis discussed |
| **Results** | | | | |
| Study parameters | 18 | Report the values, ranges, references, and if used, probability distributions for all parameters. Report reasons or sources for distributions used to represent uncertainty where appropriate. Providing a table to show the input values is strongly recommended. | NO | Distributions or uncertainty not discussed |
| Incremental costs and outcomes | 19 | For each intervention, report mean values for the main categories of estimated costs and outcomes of interest, as well as mean differences between the comparator groups. If applicable, report incremental cost-effectiveness ratios. | YES | Table costs of NP |
| Characterizing uncertainty | 20a | *Single study–based economic evaluation*: Describe the effects of sampling uncertainty for estimated incremental cost, incremental effectiveness, and incremental cost-effectiveness, together with the impact of methodological assumptions (such as discount rate, study perspective). | NO | No discussion |
|  | 20b | *Model-based economic evaluation*: Describe the effects on the results of uncertainty for all input parameters, and uncertainty related to the structure of the model and assumptions. | NA |  |
| Characterizing heterogeneity | 21 | If applicable, report differences in costs, outcomes, or cost-effectiveness that can be explained by variations between subgroups of patients with different baseline characteristics or other observed variability in effects that are not reducible by more information. | NA |  |
| **Discussion** | | | | |
| Study findings, limitations, generalizability, and current knowledge | 22 | Summarize key study findings and describe how they support the conclusions reached. Discuss limitations and the generalizability of the findings and how the findings fit with current knowledge. | YES | Cost per NP visit $316; cost per usual care $480; cost difference  -$164. Higher number of patients achieved optimal dose |
| **Other** | | | | |
| Source of funding | 23 | Describe how the study was funded and the role of the funder in the identification, design, conduct, and reporting of the analysis. Describe other nonmonetary sources of support. | YES |  |
| Conflicts of interest | 24 | Describe any potential for conflict of interest among study contributors in accordance with journal policy. In the absence of a journal policy, we recommend authors comply with International Committee of Medical Journal Editors’ recommendations. | YES |  |
| **Total score** | | | | 67% (14/21) |

**Supplementary Table 1d: Critical appraisal of Blum et al. 2020 (16)**

| **Section/item** | **Item no.** | **Recommendation** | **Reported** | **Discussion** |
| --- | --- | --- | --- | --- |
| **Title and abstract** | | | | |
| Title | 1 | Identify the study as an economic evaluation, or use more specific terms such as “cost-effectiveness analysis” and describe the interventions compared. | YES | No comparators in title |
| Abstract | 2 | Provide a structured summary of objectives, perspective, setting, methods (including study design and inputs), results (including base-case and uncertainty analyses), and conclusions. | YES | Decision analytic microsimulation model from health care perspective. |
| **Introduction** | | | | |
| Background and objectives | 3 | Provide an explicit statement of the broader context for the study. | YES |  |
|  |  | Present the study question and its relevance for health policy or practice decisions. | YES |  |
| **Methods** | | | | |
| Target population and subgroups | 4 | Describe characteristics of the base-case population and subgroups analyzed including why they were chosen. | YES | Involves patients aged over 75 years after discharged from a HF hospitalisation |
| Setting and location | 5 | State relevant aspects of the system(s) in which the decision(s) need(s) to be made. | YES | Community setting in HF |
| Study perspective | 6 | Describe the perspective of the study and relate this to the costs being evaluated. | YES |  |
| Comparators | 7 | Describe the interventions or strategies being compared and state why they were chosen. | YES | Intervention of a transitional care program comparing standard care and intensive transitional services post-discharge. Three transitional services were compared: home visits, nurse case management and disease management clinics |
| Time horizon | 8 | State the time horizon(s) over which costs and consequences are being evaluated and say why appropriate. | YES | Lifetime |
| Discount rate | 9 | Report the choice of discount rate(s) used for costs and outcomes and say why appropriate. | YES | Discount rate is 3% |
| Choice of health outcomes | 10 | Describe what outcomes were used as the measure(s) of benefit in the evaluation and their relevance for the type of analysis performed. | YES | All-cause mortality, all-cause rehospitalisations from a meta-analysis  Costs were several RCTs |
| Measurement of effectiveness | 11a | *Single study–based estimates*: Describe fully the design features of the single effectiveness study and why the single study was a sufficient source of clinical effectiveness data. | YES | QALYs were from the EPHESUS trial, a multicentre RCT of eplerenone in 6232 HF patients |
|  | 11b | *Synthesis-based estimates*: Describe fully the methods used for the identification of included studies and synthesis of clinical effectiveness data. | NA |  |
| Measurement and valuation of preference-based outcomes | 12 | If applicable, describe the population and methods used to elicit preferences for outcomes. | NA |  |
| Estimating resources and costs | 13a | *Single study–based economic evaluation*: Describe approaches used to estimate resource use associated with the alternative interventions. Describe primary or secondary research methods for valuing each resource item in terms of its unit cost. Describe any adjustments made to approximate to opportunity costs. | NA |  |
|  | 13b | *Model-based economic evaluation***:** Describe approaches and data sources used to estimate resource use associated with model health states. Describe primary or secondary research methods for valuing each resource item in terms of its unit cost. Describe any adjustments made to approximate to opportunity costs. | YES | Costs for hospitalisation were taken from a national database.  Costs of interventions were from RCTs for each intervention. |
| Currency, price date, and conversion | 14 | Report the dates of the estimated resource quantities and unit costs. Describe methods for adjusting estimated unit costs to the year of reported costs if necessary. Describe methods for converting costs into a common currency base and the exchange rate. | YES | Costs were inflation adjusted to 2018 and purchasing power parity was used to convert to US dollars |
| Choice of model | 15 | Describe and give reasons for the specific type of decision-analytic model used. Providing a figure to show model structure is strongly recommended. | YES |  |
| Assumptions | 16 | Describe all structural or other assumptions underpinning the decision-analytic model. | YES |  |
| Analytic methods | 17 | Describe all analytic methods supporting the evaluation. This could include methods for dealing with skewed, missing, or censored data; extrapolation methods; methods for pooling data; approaches to validate or make adjustments (e.g., half-cycle corrections) to a model; and methods for handling population heterogeneity and uncertainty. | YES | Uncertainty and sensitivity analyses consisted of 1-way and 2-way deterministic analyses and PSA with bootstrapping. |
| **Results** | | | | |
| Study parameters | 18 | Report the values, ranges, references, and if used, probability distributions for all parameters. Report reasons or sources for distributions used to represent uncertainty where appropriate. Providing a table to show the input values is strongly recommended. | YES | Table 1 & supplementary table 6 |
| Incremental costs and outcomes | 19 | For each intervention, report mean values for the main categories of estimated costs and outcomes of interest, as well as mean differences between the comparator groups. If applicable, report incremental cost-effectiveness ratios. | YES | Mean values of costs and outcomes presented. Mean cost differences between groups not discussed.  NHV increased costs and QALYs with an ICER: $19570 US per QALY |
| Characterizing uncertainty | 20a | *Single study–based economic evaluation*: Describe the effects of sampling uncertainty for estimated incremental cost, incremental effectiveness, and incremental cost-effectiveness, together with the impact of methodological assumptions (such as discount rate, study perspective). | NA |  |
|  | 20b | *Model-based economic evaluation*: Describe the effects on the results of uncertainty for all input parameters, and uncertainty related to the structure of the model and assumptions. | YES | Tornado diagram in supplementary material. Results of sensitivity analyses discussed |
| Characterizing heterogeneity | 21 | If applicable, report differences in costs, outcomes, or cost-effectiveness that can be explained by variations between subgroups of patients with different baseline characteristics or other observed variability in effects that are not reducible by more information. | NO | There was no discussion |
| **Discussion** | | | | |
| Study findings, limitations, generalizability, and current knowledge | 22 | Summarize key study findings and describe how they support the conclusions reached. Discuss limitations and the generalizability of the findings and how the findings fit with current knowledge. | YES | NHV was the dominant model of care with increased costs and QALYs with an ICER: $19570 US per QALY  PSA showed that transitional care services were preferred over standard care at a WTP of $50000 |
| **Other** | | | | |
| Source of funding | 23 | Describe how the study was funded and the role of the funder in the identification, design, conduct, and reporting of the analysis. Describe other nonmonetary sources of support. | YES |  |
| Conflicts of interest | 24 | Describe any potential for conflict of interest among study contributors in accordance with journal policy. In the absence of a journal policy, we recommend authors comply with International Committee of Medical Journal Editors’ recommendations. | YES |  |
| **Total score** | | | | 96% (23/24) |

**Supplementary Table 2: Economic evaluations of remote patient monitoring**

**Supplementary Table 2a: Critical appraisal of Herbert et al. 2008 (17)**

| **Section/item** | **Item no.** | **Recommendation** | **Reported** | **Discussion** |
| --- | --- | --- | --- | --- |
| **Title and abstract** | | | | |
| Title | 1 | Identify the study as an economic evaluation, or use more specific terms such as “cost-effectiveness analysis” and describe the interventions compared. | YES | No comparator in the title. No economic perspective. |
| Abstract | 2 | Provide a structured summary of objectives, perspective, setting, methods (including study design and inputs), results (including base-case and uncertainty analyses), and conclusions. | YES | Sensitivity analysis included |
| **Introduction** | | | | |
| Background and objectives | 3 | Provide an explicit statement of the broader context for the study. | YES | Covered well |
|  |  | Present the study question and its relevance for health policy or practice decisions. | YES | Covered well |
| **Methods** | | | | |
| Target population and subgroups | 4 | Describe characteristics of the base-case population and subgroups analysed including why they were chosen. | YES | HF patients from outpatient clinics and living in Harlem, NY |
| Setting and location | 5 | State relevant aspects of the system(s) in which the decision(s) need(s) to be made. | YES | HF patients from outpatient clinics and living in the community |
| Study perspective | 6 | Describe the perspective of the study and relate this to the costs being evaluated. | YES | Societal and payer perspective |
| Comparators | 7 | Describe the interventions or strategies being compared and state why they were chosen. | YES | Usual care vs nurse-led program with 1 face-to-face visits and telephone follow-up over 12 months |
| Time horizon | 8 | State the time horizon(s) over which costs and consequences are being evaluated and say why appropriate. | YES | 12 months |
| Discount rate | 9 | Report the choice of discount rate(s) used for costs and outcomes and say why appropriate. | NA | As only 12 months |
| Choice of health outcomes | 10 | Describe what outcomes were used as the measure(s) of benefit in the evaluation and their relevance for the type of analysis performed. | YES | Hospitalisations, QALYs, costs, |
| Measurement of effectiveness | 11a | *Single study–based estimates*: Describe fully the design features of the single effectiveness study and why the single study was a sufficient source of clinical effectiveness data. | YES | Economic evaluation conducted alongside an RCT. QALYs measured by SF-12 and translated into Health Utility Mark 3 and EQ-5D using methods in ref 7. |
|  | 11b | *Synthesis-based estimates*: Describe fully the methods used for the identification of included studies and synthesis of clinical effectiveness data. | NA |  |
| Measurement and valuation of preference-based outcomes | 12 | If applicable, describe the population and methods used to elicit preferences for outcomes. | YES | QALYs measured with EQ-5D done at 5 timepoints and calculating area under the curve |
| Estimating resources and costs | 13a | *Single study–based economic evaluation*: Describe approaches used to estimate resource use associated with the alternative interventions. Describe primary or secondary research methods for valuing each resource item in terms of its unit cost. Describe any adjustments made to approximate to opportunity costs. | YES | Costs estimated for intervention, medical costs and nonmedical costs but out-of-pocket cost to patient not included eg time in outpatient clinic, travel expenses  Hospitalisation costs from medicare fee schedule  Medication costs from Medicaid  Patient diaries used for inpatient and outpatient visits at other hospitals  Costs and data sources listed in Appendix |
|  | 13b | *Model-based economic evaluation***:** Describe approaches and data sources used to estimate resource use associated with model health states. Describe primary or secondary research methods for valuing each resource item in terms of its unit cost. Describe any adjustments made to approximate to opportunity costs. | NA |  |
| Currency, price date, and conversion | 14 | Report the dates of the estimated resource quantities and unit costs. Describe methods for adjusting estimated unit costs to the year of reported costs if necessary. Describe methods for converting costs into a common currency base and the exchange rate. | NO | Costs from 2005 |
| Choice of model | 15 | Describe and give reasons for the specific type of decision-analytic model used. Providing a figure to show model structure is strongly recommended. | NA |  |
| Assumptions | 16 | Describe all structural or other assumptions underpinning the decision-analytic model. | NO | Not discussed |
| Analytic methods | 17 | Describe all analytic methods supporting the evaluation. This could include methods for dealing with skewed, missing, or censored data; extrapolation methods; methods for pooling data; approaches to validate or make adjustments (e.g., half-cycle corrections) to a model; and methods for handling population heterogeneity and uncertainty. | YES | Skewed data, missing data and distributions addressed. Uncertainty addressed using 500 bootstrapped replicates. Sensitivity analysis consisted of calculating ICER for completed surveys vs missing survey data |
| **Results** | | | | |
| Study parameters | 18 | Report the values, ranges, references, and if used, probability distributions for all parameters. Report reasons or sources for distributions used to represent uncertainty where appropriate. Providing a table to show the input values is strongly recommended. | YES | Distributions not discussed  See 13a for cost references |
| Incremental costs and outcomes | 19 | For each intervention, report mean values for the main categories of estimated costs and outcomes of interest, as well as mean differences between the comparator groups. If applicable, report incremental cost-effectiveness ratios. | YES | Cost details in Table 2 and cost difference |
| Characterizing uncertainty | 20a | *Single study–based economic evaluation*: Describe the effects of sampling uncertainty for estimated incremental cost, incremental effectiveness, and incremental cost-effectiveness, together with the impact of methodological assumptions (such as discount rate, study perspective). | YES | Uncertainty was only checked on missing survey data and compared US prices vs New York prices |
|  | 20b | *Model-based economic evaluation*: Describe the effects on the results of uncertainty for all input parameters, and uncertainty related to the structure of the model and assumptions. | NA |  |
| Characterizing heterogeneity | 21 | If applicable, report differences in costs, outcomes, or cost-effectiveness that can be explained by variations between subgroups of patients with different baseline characteristics or other observed variability in effects that are not reducible by more information. | NA | Inner city population so not generaliseable |
| **Discussion** | | | | |
| Study findings, limitations, generalizability, and current knowledge | 22 | Summarize key study findings and describe how they support the conclusions reached. Discuss limitations and the generalizability of the findings and how the findings fit with current knowledge. | YES | Nurse-led program was cost-effective but it was more costly and more effective compared to UC  Inner city population so not generaliseable |
| **Other** | | | | |
| Source of funding | 23 | Describe how the study was funded and the role of the funder in the identification, design, conduct, and reporting of the analysis. Describe other nonmonetary sources of support. | YES |  |
| Conflicts of interest | 24 | Describe any potential for conflict of interest among study contributors in accordance with journal policy. In the absence of a journal policy, we recommend authors comply with International Committee of Medical Journal Editors’ recommendations. | YES |  |
| **Total score** | | | | 91% (20/22) |

**Supplementary Table 2b: Critical appraisal of Klersy et al. 2011 (22)**

| **Section/item** | **Item no.** | **Recommendation** | **Reported** | **Discussion** |
| --- | --- | --- | --- | --- |
| **Title and abstract** | | | | |
| Title | 1 | Identify the study as an economic evaluation, or use more specific terms such as “cost-effectiveness analysis” and describe the interventions compared. | YES | No comparator in the title. No economic perspective. |
| Abstract | 2 | Provide a structured summary of objectives, perspective, setting, methods (including study design and inputs), results (including base-case and uncertainty analyses), and conclusions. | YES | No uncertainty analysis |
| **Introduction** | | | | |
| Background and objectives | 3 | Provide an explicit statement of the broader context for the study. | YES | Covered well |
|  |  | Present the study question and its relevance for health policy or practice decisions. | YES | Covered well |
| **Methods** | | | | |
| Target population and subgroups | 4 | Describe characteristics of the base-case population and subgroups analysed including why they were chosen. | YES | HF patients enrolled in RCTs of telemonitoring. No demographic table |
| Setting and location | 5 | State relevant aspects of the system(s) in which the decision(s) need(s) to be made. | YES | Retrospective economic evaluation of meta-analysis of RCTs involving remote monitoring |
| Study perspective | 6 | Describe the perspective of the study and relate this to the costs being evaluated. | YES | Third party payer and only direct health care costs were included. |
| Comparators | 7 | Describe the interventions or strategies being compared and state why they were chosen. | YES | RCTs that compared telephone support approach or remote monitoring eg pacemaker, transfer of physiological data via a remote monitor vs usual care  It merged two different approaches as one intervention which is a flawed approach |
| Time horizon | 8 | State the time horizon(s) over which costs and consequences are being evaluated and say why appropriate. | YES | 1 year but some RCTs only followed up for 6 months |
| Discount rate | 9 | Report the choice of discount rate(s) used for costs and outcomes and say why appropriate. | YES | No discount or inflation |
| Choice of health outcomes | 10 | Describe what outcomes were used as the measure(s) of benefit in the evaluation and their relevance for the type of analysis performed. | YES | QALY was taken from Herbert et al study (ref 14).  Mortality taken from a recent meta-analysis (ref 5)  Hospitalisation over 12 months  It would have been more robust to take all outcomes from the same RCT |
| Measurement of effectiveness | 11a | *Single study–based estimates*: Describe fully the design features of the single effectiveness study and why the single study was a sufficient source of clinical effectiveness data. | NA |  |
|  | 11b | *Synthesis-based estimates*: Describe fully the methods used for the identification of included studies and synthesis of clinical effectiveness data. | YES | Search criteria included and databases searched. Methods were appropriate |
| Measurement and valuation of preference-based outcomes | 12 | If applicable, describe the population and methods used to elicit preferences for outcomes. | NA |  |
| Estimating resources and costs | 13a | *Single study–based economic evaluation*: Describe approaches used to estimate resource use associated with the alternative interventions. Describe primary or secondary research methods for valuing each resource item in terms of its unit cost. Describe any adjustments made to approximate to opportunity costs. | NA |  |
|  | 13b | *Model-based economic evaluation***:** Describe approaches and data sources used to estimate resource use associated with model health states. Describe primary or secondary research methods for valuing each resource item in terms of its unit cost. Describe any adjustments made to approximate to opportunity costs. | YES | See number 10. Only costs of HF hospitalisations included. No cost of intervention or outpatient visits or medications or non-HF hospitalisations. |
| Currency, price date, and conversion | 14 | Report the dates of the estimated resource quantities and unit costs. Describe methods for adjusting estimated unit costs to the year of reported costs if necessary. Describe methods for converting costs into a common currency base and the exchange rate. | NO | No conversion, cost taken from year of enrolment |
| Choice of model | 15 | Describe and give reasons for the specific type of decision-analytic model used. Providing a figure to show model structure is strongly recommended. | YES | Figure 1. Model only has HF-hospitalisation or non-HF and then cost is based on number of HF hospitalisations over 1 year. There is no reoccurring state and no mortality state in model even though text describes mortality transition state from meta-analysis |
| Assumptions | 16 | Describe all structural or other assumptions underpinning the decision-analytic model. | NO | Assumptions not discussed |
| Analytic methods | 17 | Describe all analytic methods supporting the evaluation. This could include methods for dealing with skewed, missing, or censored data; extrapolation methods; methods for pooling data; approaches to validate or make adjustments (e.g., half-cycle corrections) to a model; and methods for handling population heterogeneity and uncertainty. | YES | Sensitivity analysis compared main endpoint of hospitalisation for UC vs telemonitoring and UC vs remote monitoring. Also adjusted for length of follow-up (<6 months and >6 months)  Linear decision model developed |
| **Results** | | | | |
| Study parameters | 18 | Report the values, ranges, references, and if used, probability distributions for all parameters. Report reasons or sources for distributions used to represent uncertainty where appropriate. Providing a table to show the input values is strongly recommended. | YES | Costs for each intervention in a table. No distributions used |
| Incremental costs and outcomes | 19 | For each intervention, report mean values for the main categories of estimated costs and outcomes of interest, as well as mean differences between the comparator groups. If applicable, report incremental cost-effectiveness ratios. | YES | Table 3 lists the means and CIs and cost differences. No ICER in table. |
| Characterizing uncertainty | 20a | *Single study–based economic evaluation*: Describe the effects of sampling uncertainty for estimated incremental cost, incremental effectiveness, and incremental cost-effectiveness, together with the impact of methodological assumptions (such as discount rate, study perspective). | NA |  |
|  | 20b | *Model-based economic evaluation*: Describe the effects on the results of uncertainty for all input parameters, and uncertainty related to the structure of the model and assumptions. | YES | Sensitivity analysis compared main endpoint of hospitalisation for UC vs telemonitoring and UC vs remote monitoring. Also adjusted for length of follow-up (<6 months and >6 months) |
| Characterizing heterogeneity | 21 | If applicable, report differences in costs, outcomes, or cost-effectiveness that can be explained by variations between subgroups of patients with different baseline characteristics or other observed variability in effects that are not reducible by more information. | NA |  |
| **Discussion** | | | | |
| Study findings, limitations, generalizability, and current knowledge | 22 | Summarize key study findings and describe how they support the conclusions reached. Discuss limitations and the generalizability of the findings and how the findings fit with current knowledge. | YES | Remote patient monitoring (RPM) was dominant strategy. Savings/patient was €450. Less hospitalisations in RPM groups.  QALY gain of 0.06 |
| **Other** | | | | |
| Source of funding | 23 | Describe how the study was funded and the role of the funder in the identification, design, conduct, and reporting of the analysis. Describe other nonmonetary sources of support. | YES |  |
| Conflicts of interest | 24 | Describe any potential for conflict of interest among study contributors in accordance with journal policy. In the absence of a journal policy, we recommend authors comply with International Committee of Medical Journal Editors’ recommendations. | YES |  |
| **Total score** | | | | 91% (21/23) |

**Supplementary Table 2c: Critical appraisal of Boyne et al. 2013 (18)**

| **Section/item** | **Item no.** | **Recommendation** | **Reported** | **Discussion** |
| --- | --- | --- | --- | --- |
| **Title and abstract** | | | | |
| Title | 1 | Identify the study as an economic evaluation, or use more specific terms such as “cost-effectiveness analysis” and describe the interventions compared. | YES | No economic perspective in title. |
| Abstract | 2 | Provide a structured summary of objectives, perspective, setting, methods (including study design and inputs), results (including base-case and uncertainty analyses), and conclusions. | YES | No economic perspective. No sensitivity analysis included |
| **Introduction** | | | | |
| Background and objectives | 3 | Provide an explicit statement of the broader context for the study. | YES | Covered well |
|  |  | Present the study question and its relevance for health policy or practice decisions. | YES | Covered well |
| **Methods** | | | | |
| Target population and subgroups | 4 | Describe characteristics of the base-case population and subgroups analysed including why they were chosen. | YES | HF patients from three outpatient clinics |
| Setting and location | 5 | State relevant aspects of the system(s) in which the decision(s) need(s) to be made. | YES | HF patients from outpatient clinics and living in the community |
| Study perspective | 6 | Describe the perspective of the study and relate this to the costs being evaluated. | YES | Societal and payer perspective |
| Comparators | 7 | Describe the interventions or strategies being compared and state why they were chosen. | YES | Usual care vs telemonitoring. Usual care group had 4 outpatient clinic visits over 12 months. Telemonitoring group had 2 outpatient clinic visits and the telemonitoring system with daily follow-up of symptoms and knowledge |
| Time horizon | 8 | State the time horizon(s) over which costs and consequences are being evaluated and say why appropriate. | YES | 12 months |
| Discount rate | 9 | Report the choice of discount rate(s) used for costs and outcomes and say why appropriate. | NA | As only 12 months |
| Choice of health outcomes | 10 | Describe what outcomes were used as the measure(s) of benefit in the evaluation and their relevance for the type of analysis performed. | YES | Hospitalisations, QALYs, and costs |
| Measurement of effectiveness | 11a | *Single study–based estimates*: Describe fully the design features of the single effectiveness study and why the single study was a sufficient source of clinical effectiveness data. | YES | Economic evaluation conducted alongside an RCT. QALYs measured by EQ-5D at 3,6 and 12 months |
|  | 11b | *Synthesis-based estimates*: Describe fully the methods used for the identification of included studies and synthesis of clinical effectiveness data. | NA |  |
| Measurement and valuation of preference-based outcomes | 12 | If applicable, describe the population and methods used to elicit preferences for outcomes. | YES | QALYs measured with EQ-5D done at 3,6 and 12 months |
| Estimating resources and costs | 13a | *Single study–based economic evaluation*: Describe approaches used to estimate resource use associated with the alternative interventions. Describe primary or secondary research methods for valuing each resource item in terms of its unit cost. Describe any adjustments made to approximate to opportunity costs. | YES | Costs were recorded in a cost diary for GP visits, telephone and face-to-face contacts with MD team.  Medication costs from Dutch Pharmacotherapeutic Compass. Hospital procedure costs from hospital financial departments.  Readmission, ED visits, GP visits, and MD team visits were taken from a national cost manual. |
|  | 13b | *Model-based economic evaluation***:** Describe approaches and data sources used to estimate resource use associated with model health states. Describe primary or secondary research methods for valuing each resource item in terms of its unit cost. Describe any adjustments made to approximate to opportunity costs. | NA |  |
| Currency, price date, and conversion | 14 | Report the dates of the estimated resource quantities and unit costs. Describe methods for adjusting estimated unit costs to the year of reported costs if necessary. Describe methods for converting costs into a common currency base and the exchange rate. | YES | Costs from 2008 in Euros |
| Choice of model | 15 | Describe and give reasons for the specific type of decision-analytic model used. Providing a figure to show model structure is strongly recommended. | NA |  |
| Assumptions | 16 | Describe all structural or other assumptions underpinning the decision-analytic model. | NA |  |
| Analytic methods | 17 | Describe all analytic methods supporting the evaluation. This could include methods for dealing with skewed, missing, or censored data; extrapolation methods; methods for pooling data; approaches to validate or make adjustments (e.g., half-cycle corrections) to a model; and methods for handling population heterogeneity and uncertainty. | YES | Skewed data, missing data and distributions addressed. Uncertainty addressed using 1000 bootstrapped replicates for confidence intervals. |
| **Results** | | | | |
| Study parameters | 18 | Report the values, ranges, references, and if used, probability distributions for all parameters. Report reasons or sources for distributions used to represent uncertainty where appropriate. Providing a table to show the input values is strongly recommended. | NO | Distributions not discussed |
| Incremental costs and outcomes | 19 | For each intervention, report mean values for the main categories of estimated costs and outcomes of interest, as well as mean differences between the comparator groups. If applicable, report incremental cost-effectiveness ratios. | YES | Cost details in Table 2 and cost difference |
| Characterizing uncertainty | 20a | *Single study–based economic evaluation*: Describe the effects of sampling uncertainty for estimated incremental cost, incremental effectiveness, and incremental cost-effectiveness, together with the impact of methodological assumptions (such as discount rate, study perspective). | NO | The effects of uncertainty was not discussed |
|  | 20b | *Model-based economic evaluation*: Describe the effects on the results of uncertainty for all input parameters, and uncertainty related to the structure of the model and assumptions. | NA |  |
| Characterizing heterogeneity | 21 | If applicable, report differences in costs, outcomes, or cost-effectiveness that can be explained by variations between subgroups of patients with different baseline characteristics or other observed variability in effects that are not reducible by more information. | YES | Subgroup analyses of duration of HF and separate analyses of ICER for each site was conducted |
| **Discussion** | | | | |
| Study findings, limitations, generalizability, and current knowledge | 22 | Summarize key study findings and describe how they support the conclusions reached. Discuss limitations and the generalizability of the findings and how the findings fit with current knowledge. | YES | There were no significant differences in annual costs per patient between groups. At a threshold of E50,000 the probability of telemonitoring being cost-effective was 48%. |
| **Other** | | | | |
| Source of funding | 23 | Describe how the study was funded and the role of the funder in the identification, design, conduct, and reporting of the analysis. Describe other nonmonetary sources of support. | YES |  |
| Conflicts of interest | 24 | Describe any potential for conflict of interest among study contributors in accordance with journal policy. In the absence of a journal policy, we recommend authors comply with International Committee of Medical Journal Editors’ recommendations. | NO |  |
| **Total score** | | | | 86% (18/21) |

**Supplementary Table 2d: Critical appraisal of Thokala et al. 2013 (15)**

| **Section/item** | **Item no.** | **Recommendation** | **Reported** | **Discussion** |
| --- | --- | --- | --- | --- |
| **Title and abstract** | | | | |
| Title | 1 | Identify the study as an economic evaluation, or use more specific terms such as “cost-effectiveness analysis” and describe the interventions compared. | YES | No comparator in the title. No economic perspective. |
| Abstract | 2 | Provide a structured summary of objectives, perspective, setting, methods (including study design and inputs), results (including base-case and uncertainty analyses), and conclusions. | YES | No uncertainty analysis |
| **Introduction** | | | | |
| Background and objectives | 3 | Provide an explicit statement of the broader context for the study. | YES | Covered well |
|  |  | Present the study question and its relevance for health policy or practice decisions. | YES | Covered well |
| **Methods** | | | | |
| Target population and subgroups | 4 | Describe characteristics of the base-case population and subgroups analysed including why they were chosen. | YES | Patients post-discharge from a HF admission. Enrolled within 30 days post-discharge. |
| Setting and location | 5 | State relevant aspects of the system(s) in which the decision(s) need(s) to be made. | YES | Retrospective economic evaluation of a network meta-analysis using secondary data sources |
| Study perspective | 6 | Describe the perspective of the study and relate this to the costs being evaluated. | YES | NHS in England and Wales |
| Comparators | 7 | Describe the interventions or strategies being compared and state why they were chosen. | YES | Network meta-analysis compared telephone support over the phone, structured telephone support via a machine or telemonitoring with transmitted data vs usual care (ref 11) |
| Time horizon | 8 | State the time horizon(s) over which costs and consequences are being evaluated and say why appropriate. | YES | 30 year. Not appropriate due to high mortality |
| Discount rate | 9 | Report the choice of discount rate(s) used for costs and outcomes and say why appropriate. | YES | Discount of 3.5% which is appropriate. |
| Choice of health outcomes | 10 | Describe what outcomes were used as the measure(s) of benefit in the evaluation and their relevance for the type of analysis performed. | YES | QALYs, hospitalisations taken from network meta-analysis, mortality |
| Measurement of effectiveness | 11a | *Single study–based estimates*: Describe fully the design features of the single effectiveness study and why the single study was a sufficient source of clinical effectiveness data. | NA |  |
|  | 11b | *Synthesis-based estimates*: Describe fully the methods used for the identification of included studies and synthesis of clinical effectiveness data. | YES | No details about network meta-analysis (ref 11) or search strategy |
| Measurement and valuation of preference-based outcomes | 12 | If applicable, describe the population and methods used to elicit preferences for outcomes. | NA |  |
| Estimating resources and costs | 13a | *Single study–based economic evaluation*: Describe approaches used to estimate resource use associated with the alternative interventions. Describe primary or secondary research methods for valuing each resource item in terms of its unit cost. Describe any adjustments made to approximate to opportunity costs. | NA |  |
|  | 13b | *Model-based economic evaluation***:** Describe approaches and data sources used to estimate resource use associated with model health states. Describe primary or secondary research methods for valuing each resource item in terms of its unit cost. Describe any adjustments made to approximate to opportunity costs. | YES | Average number of monthly rehospitalisations from network meta-analysis.  Probability of mortality from CHARM study  QALY taken from 4 studies (ref 13-16) for UC and for RPM disutility was taken from Yao (ref 17) |
| Currency, price date, and conversion | 14 | Report the dates of the estimated resource quantities and unit costs. Describe methods for adjusting estimated unit costs to the year of reported costs if necessary. Describe methods for converting costs into a common currency base and the exchange rate. | NO | Not discussed |
| Choice of model | 15 | Describe and give reasons for the specific type of decision-analytic model used. Providing a figure to show model structure is strongly recommended. | NO | Not discussed |
| Assumptions | 16 | Describe all structural or other assumptions underpinning the decision-analytic model. | NO | Assumed all interventions went for 6 months and then probability of hospitalisations changed back to same at baseline. Heterogeniety of studies in network meta-analysis |
| Analytic methods | 17 | Describe all analytic methods supporting the evaluation. This could include methods for dealing with skewed, missing, or censored data; extrapolation methods; methods for pooling data; approaches to validate or make adjustments (e.g., half-cycle corrections) to a model; and methods for handling population heterogeneity and uncertainty. | YES | Half-cycle corrections included. PSA done with 10 000 iterations using different estimates for the risks, HR, costs and utilities and different thresholds of WTP. |
| **Results** | | | | |
| Study parameters | 18 | Report the values, ranges, references, and if used, probability distributions for all parameters. Report reasons or sources for distributions used to represent uncertainty where appropriate. Providing a table to show the input values is strongly recommended. | YES | See 13b |
| Incremental costs and outcomes | 19 | For each intervention, report mean values for the main categories of estimated costs and outcomes of interest, as well as mean differences between the comparator groups. If applicable, report incremental cost-effectiveness ratios. | YES | Cost of intervention initial visit, cost of UC and cost of hospitalisation. Costs included in table 4 and 5 |
| Characterizing uncertainty | 20a | *Single study–based economic evaluation*: Describe the effects of sampling uncertainty for estimated incremental cost, incremental effectiveness, and incremental cost-effectiveness, together with the impact of methodological assumptions (such as discount rate, study perspective). | NA |  |
|  | 20b | *Model-based economic evaluation*: Describe the effects on the results of uncertainty for all input parameters, and uncertainty related to the structure of the model and assumptions. | YES | Uncertainty analysis using different estimates of disutility for hospitalisation and length of follow-up for costing of interventions produced similar results to base case analysis |
| Characterizing heterogeneity | 21 | If applicable, report differences in costs, outcomes, or cost-effectiveness that can be explained by variations between subgroups of patients with different baseline characteristics or other observed variability in effects that are not reducible by more information. | NA |  |
| **Discussion** | | | | |
| Study findings, limitations, generalizability, and current knowledge | 22 | Summarize key study findings and describe how they support the conclusions reached. Discuss limitations and the generalizability of the findings and how the findings fit with current knowledge. | YES | Telemonitoring is most cost-effective. Telemonitoring had ICER of €11 873/QALY and telephone support had an ICER of €228 035 |
| **Other** | | | | |
| Source of funding | 23 | Describe how the study was funded and the role of the funder in the identification, design, conduct, and reporting of the analysis. Describe other nonmonetary sources of support. | YES |  |
| Conflicts of interest | 24 | Describe any potential for conflict of interest among study contributors in accordance with journal policy. In the absence of a journal policy, we recommend authors comply with International Committee of Medical Journal Editors’ recommendations. | YES |  |
| **Total score** | | | | 86% (19/22) |
